# Comparing Machine Learning Classifiers and Linear/Logistic Regression to Explore the Relationship between Hand Dimensions and Demographic Characteristics

**DOI:** 10.1371/journal.pone.0165521

**Published:** 2016-11-02

**Authors:** Oscar Miguel-Hurtado, Richard Guest, Sarah V. Stevenage, Greg J. Neil, Sue Black

**Affiliations:** 1 School of Engineering and Digital Arts, University of Kent, Canterbury, United Kingdom; 2 Department of Psychology, University of Southampton, Southampton, United Kingdom; 3 Centre for Anatomy and Human Identification, University of Dundee, Dundee, United Kingdom; National University of Defense Technology, CHINA

## Abstract

Understanding the relationship between physiological measurements from human subjects and their demographic data is important within both the biometric and forensic domains. In this paper we explore the relationship between measurements of the human hand and a range of demographic features. We assess the ability of linear regression and machine learning classifiers to predict demographics from hand features, thereby providing evidence on both the strength of relationship and the key features underpinning this relationship. Our results show that we are able to predict sex, height, weight and foot size accurately within various data-range bin sizes, with machine learning classification algorithms out-performing linear regression in most situations. In addition, we identify the features used to provide these relationships applicable across multiple applications.

## 1 Introduction

Automated Information and Communications Technology (ICT)-based biometric technologies that recognise humans through physiological or behavioural characteristics enable a convenient, accurate and repeatable method for identity assessment [1]. Using individual modalities such as face, iris, hand and voice, numerous deployments have been made in application areas such as border and physical access control, where the task is to verify an identity against a pre-enrolled template or identifying from a dataset of pre-enrolled subjects. This is similar to the task as it exists in the field of forensics, where forensic verification or identification of subjects is required by human inspection typically for legal purposes. Both disciplines require an accurate assessment of human characteristics thereby allowing confidence in the result. In the forensic domain it is frequently the case that identifications are based on multiple sources of partial evidence, therefore understanding how one characteristic of human identity maps to a further characteristic aids identification [2].

For automated biometrics, meta-data or soft-biometrics refers to additional characteristics of the subject that can be used to support or sharpen an identification or verification decision [3]. Consider an environment in which a facial system is providing primary biometric evidence. Knowing the sex of the subject enables confirmation of a primary verification decision, or allows a narrowing of a watch-list identification search space, potentially improving the certainty of an assessment, or the time required to reach a decision. Although soft-biometric traits lack distinctiveness to identify an individual, it has been shown that a combination of multiple soft-biometrics can add a valuable degree of certainty to primary verification decisions. These results are based on the ability to use known information (i.e. a hand image) to predict other previously unknown pieces of information (such as individual’s sex) [4]. These predicted pieces of information may be critical for supporting decisions within both biometric and forensic domains. This work aims to predict a complete set of soft-biometric traits from hand images: sex, height, weight and foot size, thereby providing flexibility within available data.

The human hand offers a rich seam of anatomical characteristics that are easily obtainable and measurable [5]. Furthermore, hand length measures do not require invasive techniques to collect, and they can even be collected covertly. Forensic analysis has proved the capacity to match a hand image from crime scene footage to a constrained image even under conditions that are known to impair performance with other biometrics [6]. These capacities could be enhanced by adding predicted soft-biometric traits to the decision. Alongside specific elements such as fingerprints (anatomical) and writing (behavioural), the size and geometry of the hand can lead to important demographical indicators such as sex, height, weight and foot size as soft-biometrics. These can all be used to enhance the recognition process, reduce the search space and support higher level of confidence [7]. Previous studies on hand measurement characteristics have commonly used linear regression approach when modelling the relationship between demographics and hand features [8–10]. These models have shown a reasonable level of accuracy for the prediction of height, foot size and sex. As an alternative, in [7] the authors employed machine learning classification algorithms along with sophisticated hand shape features to predict sex. However, this study sits alone in terms of its approach.

Building upon [7] we wish to extend the use of machine learning classification algorithms for the prediction of soft-biometrics from hand features, with a focus particularly on the achievable accuracy and optimal resolution of the characteristics that can be predicted. The work presented in this paper widens the use of machine learning techniques, to date used only for sex prediction, to allow prediction of other three soft-biometric traits, namely: height, weight and foot size.

We explore in this work the use of height, weight and foot size as categorical outputs. In order to obtain the benefits of soft-biometrics (i.e. narrow search space, improve assessment certainty, reduce computational time and load) it is not mandatory to use a precise prediction of these traits—as such it is suitable to have a binning classification such as low/medium/high. Taking this into account, machine learning classification algorithms are analysed for the prediction of the aforementioned soft-biometric traits.

In previous works, sex prediction from hand lengths has been investigated using logistic regression while the relationship between hand length and height/foot size have been commonly analysed through multilinear regressions. The authors are unaware of any studies analysing the relationship between hand lengths and weight. This current work represents, to the best of the authors’ knowledge, the first attempt to jointly analyse the prediction of these four soft-biometrics from hand features. Furthermore, it also represents the first attempt to use machine learning classification algorithms for the prediction of height, weight and foot size.

The main research question here is thus whether machine learning classification algorithms can improve the soft-biometric predictions obtained over-and-above the more commonly used linear/logistic regression approach. As a secondary issue, we wish to explore which classifier algorithm provides the most optimal outcome. In this regard, it is known that no single classifier outperforms others across a range of data. Acknowledging this issue, several different classifiers have been tested here in order to identify an optimal fit when predicting a specific demographic trait. Furthermore, our experiments will explore which, of a range of hand features, are most valuable when predicting each demographic trait.

This exploratory analysis is made possible due to the existence of the SuperIdentity Stimulus Database (SSD) collected as part of the SuperIdentity project [4]. This includes, amongst others biometric modalities, hand images and demographic traits such as sex, height, weight and foot size. The use of machine learning classification algorithms means that predicted continuous variables such as height or weight need to be converted into nominal attributes. As an exploratory analysis, simple binning has been used in implementation. Within our analysis we shall also use features from both left and right hands, again allowing us to explore the differences in predictive power and the relationship to symmetry. We shall also utilise our own meta-data within a pathway of analysis—by applying a-priori knowledge of sex of the subject we can explore whether other demographic elements can be predicted with greater accuracy in comparison with the situation where this information is not known.

In performing this investigation our aim is to provide evidence to a wide range of communities as to the relationships (and importantly the strength of these relationships) that can be discovered between hand measurements and demographic characteristics. These relationships can be utilised in a practical manner to assist with integration within a biometric implementation and provides evidentiary value of forensic relationships. Furthermore, the public availability of the dataset used in this work ensures repeatability and encourages further research in this area.

## 2 The relationship between hands and demographical details

Whilst the use of hand features to predict demographic traits has been widely applied in fields such as forensic science, psychology and anthropology, it currently does not have wide application in the biometric field. Across these domains the prediction of demographic traits can have several practical applications: it can improve the performance of biometric algorithms, and can reduce the search of large biometric databases decreasing the computational time for identification. It can also support re-identification in surveillance scenarios and disaster victim identification. In this work, we analyse the prediction of four key soft-biometric traits (sex, height, weight and foot size). Each have been coded as categorical values using machine learning classification algorithms, in order to assess whether this approach outperforms the more common approach of continuous value prediction by regression techniques.

In the following subsections, we provide an overview of the state of the art in the prediction of demographic traits from hand features.

### 2.1 Sex prediction

Sex prediction is one of the most studied soft-biometric traits with predictive values explored across a range of different biometric modalities such as fingerprints [11], face [12], iris [13] and hand [7]. Likewise, sex prediction has been thoroughly studied in forensic [2], anthropological [14, 15] and psychological fields [16, 17]. To provide context to our current work, we review a range of previous methodologies and assessments enabling sex prediction from hand features.

Case and Ross [18] performed a discriminant function analysis using stepwise feature selection to investigate the predictive capacity of hand features (metacarpal and phalangeal lengths extracted from hand bones) from both right and left hands. The sample was obtained from the Terry Collection [19] and contained 123 females and 136 males. The cross-validation method (n-1 out of n observations) was performed to classify observations by sex. The best sex predictors were obtained from a subset of phalangeal lengths, and were different for the left hand (85.7% success rate) and the right hand (84.3%).

In [15] the authors also analysed the use of hand features obtained from metacarpals hand bones, but including a wider set of features: total length and mid-diameter of each bone, as well as width and height of both bone ends. These measures were taken separately from both the right and left hand, and a complete set of measures were obtained for 196 subjects (118 males, 78 females). Binary logistic regression equations were calculated for sex prediction, in order to find the best combination of features to predict sex. From the right hand features, the best accuracy rate obtained was 89.3% based on three measurements from the 5^th^ metacarpal. From left hand features, the best accuracy rate was 89.8% based on three measurements of the 1^st^ metacarpal.

In [8] the authors analysed the discriminative power of three different hand features: i) hand length, ii) palm length and iii) hand breadth. This study was conducted on a sample of 500 Indian participants (230 male and 270 female) obtaining an accuracy rate of 87% for males and 91% for females based on hand breath and using data from the right hand, and 89% (for males) and 92% (for females) from the left hand based on hand breath (males) and palm length (female). These results led to an overall success rate of 89% and 90% for right and left hands respectively. The accuracy was based on a threshold named “sectioning point” Eq (1) tested on the same sample from which it was derived.

$$Sectioning\;point=\frac{Mean\;male\;value+Mean\;female\;value}{2}$$ (1)

More recently, Jee et al. [20] analysed a total of 29 hand features including length, breadth, thickness and circumference of fingers, palm and wrist in order to predict the sex of 321 subjects (167 males and 154 females). This study also investigated the influence of age range on the sex prediction accuracy. The results of this work showed a sex prediction accuracy of 90%. The model used was obtained using stepwise discriminant analysis and it included palm length, maximum hand thickness and hand breadth as sex predictor variables. The accuracy of the method was confirmed using a cross validation methodology.

A study by Amayeh et al. [7] represents the only identified work in the biometric domain using right hand features from 20 males and 20 females subjects to obtain sex prediction as a soft biometric. In this study sex was predicted from a range of hand features using image processing techniques and three different machine learning classifiers: minimum distance, k-nearest neighbours and linear discriminant analysis. The images were pre-processed to obtain the silhouette of the palm and each finger. From these six different silhouettes MPEG-7 shape descriptors (specifically Fourier-based descriptors and shape representation Zernike moments) [21] were used to represent their geometry. The highest accuracy rate (98%) was obtained by score-level fusion using MPEG-7 Fourier descriptors as the hand features and linear discriminant analysis as the classifier. The evaluation was carried out using cross-validation based on the leave-one-out approach.

Table 1 summarises the accuracy rates obtained from the different authors, along with details of the database used (country and number of participants) and the features analysed. As a whole, accuracy of sex prediction is typically around 90%, and appears to be marginally better from the left than the right hand characteristics.

**Table 1:** Sex predictions from hand lengths.

First Author	Year	Ref.	#Participants	Features	Accuracy Rate
T. Case	2007	[18]	259 (136M, 123F)	Phalange features	85.70%
G. Amayeh	2008	[7]	40 (20M, 20F)	MPEG-7 Hand shape features	98.00%
T. Kanchan	2009	[8]	500 (230M, 270F)	Hand breadth	90.00%
P. Khanpetch	2012	[15]	196 (118M, 78F)	Metacarpal features	89.80%
S. Jee	2015	[20]	321 (167M, 154F)	Palm length, hand breadth and hand thickness.	90.00%

### 2.2 Height (stature) prediction

Similar to sex prediction, there is a large literature on height prediction from hand features. However, this research comes mostly from the forensic and anthropological fields. Height prediction is of special interest within these fields due to its use in forensic examination, anthropological studies and disaster victim identification scenarios. In the biometric field, height has been proposed as a soft-biometric feature to improve gait-based systems [22, 23], however the use of hands for height prediction has hitherto been unexplored.

In 1978 Musgrave and Harneja [24] produced an analysis of the capability of using hand features to predict height. In this work the metacarpal lengths were extracted from X-ray images from both right and left hands of 120 male and 46 female subjects. These lengths were analysed using linear regression from each metacarpal. The best linear regression models showed a root mean squared error (RMSE) of 5.49cm for males and 4.70cm for females, with both models obtained from left metacarpal lengths (adjusted R-squared values were not reported).

More recent studies have used the hand length and/or hand breadth features to create linear regression models to predict height. In [10] left and right hands and foot lengths were used independently to predict height. They analysed a sample of 80 male and 75 female Turkish participants. Using only hand length and applying a linear regression approach to create linear models, this study proposed three different models for males, females and the whole sample. These models show an adjusted R-squared value of 0.52 and 4.26cm RMSE for the males, adjusted R-squared value of 0.49 and 3.49cm RMSE for females and adjusted R-squared value of 0.76 and 4.59cm RMSE for the whole combined sample.

In [9] Agnihotri et al. used a similar approach as in [10] but added hand breadth to improve height prediction. This study was conducted on 250 participants (125 male and 125 female). A linear regression approach was used to create the models to predict height for male and female samples. For males, the addition of hand breadth improved the results obtaining a linear regression model with an adjusted R-squared value of 0.39 and 4.80cm RMSE. For females, the best model used only the hand length, obtaining an adjusted R-squared value of 0.54 and 4.16cm RMSE.

Habib et al. [25] presented an examination of height prediction using the hand length plus the phalangeal lengths (excluding thumb phalanges) from both right and left hands. Multiple linear regression analysis using stepwise feature selection technique was conducted on a sample composed of 82 male and 77 female Egyptian participants. The results showed that the addition of phalangeal lengths improved the prediction within the female group, obtaining a multiple linear regression model with an adjusted R-squared value of 0.32 and 4.54cm RMSE. For the male sample the best linear regression model only included the hand length, which had an adjusted R-squared value of 0.49 and 5.30cm RMSE.

Jee et al. [26] used the same 29 hand features and population as in [20] to analyse the relationship between hand features and height using multilinear regression and stepwise feature selection. The results showed that length related variables (hand length, palm length) had higher estimation accuracy than breadth related features (hand breadth and maximum hand breadth) and thickness related features (hand thickness and maximum hand thickness). The best multilinear regression model for male height prediction showed a R-squared value of 0.425 and 4.81cm RMSE. The best female model showed a R-squared value of 0.418 and 5.08cm RMSE. Finally, the best model for both male and female population (whole sample population) presented a R-squared value of 0.642 and 5.72cm of RMSE.

The results detailed above are summarised in Table 2. The adjusted R-squared (Adj. R^2^) and the RMSE provide a statistical measure of the goodness of fit of the model and the error of the predictions. The subscript indicates whether the model was created for males (M), females (F) or for the whole sample (W).

**Table 2:** Height predictions from hand lengths.

First Author	Year	Ref.	#Participants	Regression Model	Adj. R^2^	RMSE (cm)
J. H. Musgrave	1978	[24]	166 (120M, 46F)	H_M_ ∼ Left Met1	NR	5.49 (M)
				H_F_ ∼ Left Met2	NR	4.70 (F)
S. G. Sanli	2005	[10]	155 (80M, 75F)	H_FMW_ ∼ HL	0.52 (M)	4.26 (M)
					0.49 (F)	3.49 (F)
					0.76 (W)	4.59 (W)
A. K. Agnihotri	2008	[9]	250 (125M, 135F)	H_M_ ∼ HL + HB	0.39(M)	4.80 (M)
				H_F_ ∼ HL	0.54(F)	4.16 (F)
S. R. Habib	2010	[25]	159	H_M_ ∼ HL	0.49 (M)	5.30 (M)
				H_F_ ∼ HL + PL	0.32 (F)	4.54 (F)
S. Jee	2015	[26]	321 (167M,154F)	H_M_ ∼ HL + PL + PalmL	0.42 (M)	4.81 (M)
				H_F_ ∼ HL + PL + MHB	0.42 (F)	5.08 (F)
				H_W_ ∼ WC + PL + PalmL	0.64 (W)	5.72 (W)

M = Male, F = Female, W = Both male and female

HL = Hand Length, HB = Hand Breadth, PL = Phalange lengths Met = Metacarpal

H = Height, WC = wrist circumference, MHB = Maximum hand breadth, PalmL = Palm length

NR = Not Reported

### 2.3 Foot size prediction

The prediction of foot size from hand features has not attracted much attention from the research community. However, as aforementioned, every piece of information that can be added to an identification task has the potential to improve the degree of certainty. Moreover, foot size is common evidence found in forensic investigations. The most relevant work [27] was conducted on 120 male and 120 female subjects from North India. In this work, the hand length and hand breadth were used as predictors to create a multilinear regression model to estimate foot length. The results show that the right foot length can be predicted from the right hand length and breadth with a linear model which has an adjusted R-squared value of 0.59 and 0.76cm RMSE. For the left foot length the linear model, based on left hand length and breadth, has an adjusted R-squared value of 0.64 and 0.72cm RMSE.

### 2.4 Weight prediction

Despite the fact that in 2004 the utilisation of weight was proposed as a soft-biometric [3], we were unable to locate any studies showing its prediction from hand features or other related biometric modalities. Attempts have been made, however, to use weight as a soft-biometric to improve fingerprint identification systems [28].

### 2.5 State of the art summary

The literature review for the prediction of sex and height from hand measurements indicates the interest of the research community in these areas. These two soft-biometric attributes have also been demonstrated to be of interest for the biometric community. Nevertheless, the literature review also showed that the use of machine learning classification algorithms has not yet been explored. More generally, the relationships between hand measurements and other soft-biometric measures such as foot size and weight predictions have not been a focus for many previous studies. However, as indicated in [3], the use of several soft-biometric cues simultaneously can lead to an improvement in biometric systems. For these reasons, this works aims to explore the predictions of these four key demographic traits (sex, height, foot size and weight) from hand images using machine learning classification algorithms.

It is also important to highlight the variation of these demographic traits across different ethnicities. Studies have revealed that the relationships between body measurements/features vary between populations and ethnicity [29]. The fact that most of the datasets used in the literature review articles are not publicly available, or are difficult to obtain, impedes an analysis of how these population variations can be incorporated in enhanced demographic prediction models. The authors strongly believe that the public availability of additional datasets could lead to a much better understanding of the relationships analysed in this work and, moreover, its dependencies across different populations groups.

The dataset used in this work has been made publicly available at [30], including right and left hand images, right and left hand extracted measurements and the four demographic traits from 112 participants. This public availability facilitates reproducible results, which will enable a comparison with further research in these areas. We hope that the release of this dataset will lead to the release of further datasets collected under comparable conditions with participants from different populations groups. This will boost the understanding and accuracy of the soft-biometric predictions from hand features, and their deployment on new biometric and forensic applications.

## 3 Methodology

Our study used a subcorpus of the the SuperIdentity Stimulus Database (SSD) [4] which is a multimodality dataset including: face, iris, fingerprint, hand, signature, gait and voice. The hand subcorpus contains both hand images and demographic information on the subjects and has been made publicly available [30]. This hand dataset includes both left and right hand geometry images from 112 participants along with two Excel files containing a series of length measurement (21 features, see Section 3.1 for further details) manually extracted from each image and the participant’s demographic information (sex, height, weight and foot size). The data collection exercise was given full approval independently by the University of Southampton Ethics Committee, the University of Kent Sciences Ethics Committee and the University of Dundee Research Ethics Committee. Subjects provided their written informed consent to participate in the study, using a consent procedure approved by the all three Ethics Committees.

**Fig 1:**
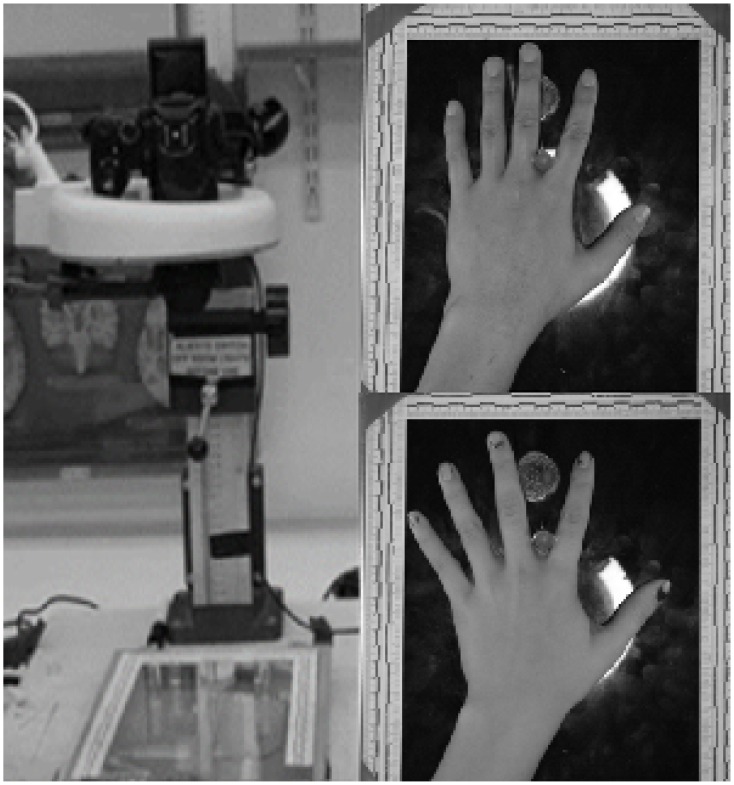
Hand camera rig and hand samples.

The SSD hand subcorpus comprises 112 participants (56 males and 56 females) restricted to Caucasians who spoke English as a first language and were aged between 18 and 35 years. Hand geometry images were captured using a Nikon D200 SLR camera, with both the palm of the hand and camera facing downwards. Participants placed each hand on an acetate sheet with a series of positioning pegs. Once the hand was correctly placed, three consecutive photographs were taken of each hand resulting in six images per subject. Fig 1 shows the rig used to capture images along with two hand image samples. Demographic information comprising data such as age (in years), sex (male or female), handedness (left, right or ambidextrous), height (in cm), weight (in kg) and foot size (in UK sizes) was also collected from each participant. Demographic information was self-reported and collected via an online survey.

### 3.1 Hand features

**Fig 2:**
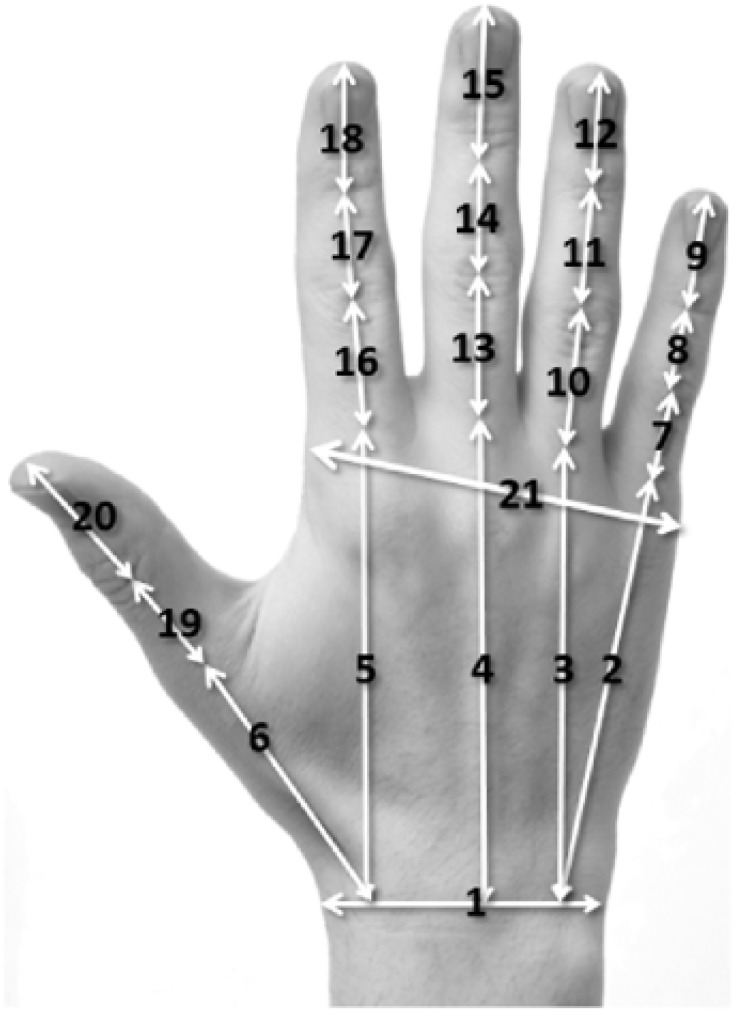
Hand lengths.

A series of length measurements (based on the underlying skeleton of the hand) were manually extracted from the first of the three images captured from both left and right hands (Fig 2).

Table 3 details all the measures extracted from each hand.

**Table 3:** Hand feature set.

#	Description	#	Description
1	Wrist Breadth	12	Ring Distal Phalanx
2	Wrist To Little	13	Middle Proximal Segment
3	Wrist To Ring	14	Middle Intermediate Phalanx
4	Wrist To Middle	15	Middle Distal Phalanx
5	Wrist To Index	16	Index Proximal Phalanx
6	Wrist To Thumb	17	Index Intermediate Phalanx
7	Little Proximal Phalanx	18	Index Distal Phalanx
8	Little Intermediate Phalanx	19	Thumb Proximal Phalanx
9	Little Distal Phalanx	20	Thumb Distal Phalanx
10	Ring Proximal Phalanx	21	Hand breadth
11	Ring Intermediate Phalanx		

### 3.2 Demographical descriptive statistics

Table 4 shows the descriptive statistics: mean, standard deviation (SD), maximum (Max) and minimum (Min) values of the four demographic traits analysed in this work. These statistics are presented separately for male, female and the entire/”whole” populations. For demographic histogram distributions please refer to S1 Fig in the Supporting Information Section.

**Table 4:** Demographic traits descriptive statistics.

Demographic	Male				Female				Whole			
	***Mean***	***SD***	***Max***	***Min***	***Mean***	***SD***	***Max***	***Min***	***Mean***	***SD***	***Max***	***Min***
Height (cm)	178	8.34	155	193	166	7.44	145	186	172	9.65	145	193
Weight (kg)	73	10.16	56	104	61	11.88	43	115	67	12.48	43	115
Log-Weight (log-kg)	4.28	0.13	4.02	4.64	4.10	0.17	3.76	4.74	4.18	0.18	3.76	4.74
Foot size (UK units)	9.5	1.57	4	13	5.6	1.30	3	9	7	2.44	3	13

The weight distributions show positive skewness (S1 Fig), where low-values have higher frequencies than high-values. Skew distributions can cause problem with machine learning classifiers due to the assumption of normal distribution. In order to avoid these problems a log-transformation of the weight was performed to obtain symmetric distributions.

### 3.3 Binning categorisation

Our experiments divided the sample into a series of discrete bins based on subject height, weight and shoe size. Obviously the more bins used, the finer the resolution of data range contained in each bin.

The categorisation of demographic traits has been performed using simple discretization (equal-width bins). This process divides the full range of the demographic traits into N intervals of equal width. These N intervals are used as categories for the specific demographic trait. The greater the number of categories used, the better resolution for the predictions. However, a high number of categories can affect the performance of the machine learning classifiers. As the number of categories increases, so does the difficulty of the classification problem. Equally, there are fewer observations for each particular category on which to train the classifier. Hence, normally a higher number of categories leads to lower classifier accuracies.

In order to analyse the impact of the different number of bins on the performance of the machine learning classifiers, three options have been used: 3, 5 and 7 bins, by means of simple discretisation. This discretisation has been performed independently for each population dataset (male, female and whole populations).

As a result of the use of simple discretisation using equal bin width, bins show different frequencies (see S2 Fig in the Supporting Information Section). In some cases, the number of bin observations are significantly different. These frequency differences reveal an unbalanced dataset and can have an impact on the machine learning classification performance.

### 3.4 Feature selection and model validation

Following hand feature extraction and demographic trait binning, a feature selection step was performed to select the most relevant features for predicting the demographic trait categories in each case. The hand features dataset was split into training and testing datasets. The training dataset contains 60% of the participants (67 out of 112) and was used to perform the feature selection.

The feature selection method applied was the “best-first search” algorithm [31]. This feature selection algorithm searched the attribute space greedily in one of three possible directions: forward, backward or bidirectional. Our experimentation used all three directions independently. The three resulting feature subsets, one for each search direction, were evaluated for each classifier, population dataset and right and left hand features. The feature selection was carried out using the WEKA machine learning engine [32] using the “Classifier subset evaluator” in combination with the “BestFirst” attribute selection implementation. The four machine learning classifiers as described in Section 3.5 were used within the “Classifier subset evaluator”. The default settings were used within the evaluator. The “BestFirst” search method was also used with its default values, however three different direction options: forward, backward or bidirectional were analysed.

Using the selected features by the “best-first search” algorithm, machine learning models were created for the three different participant groups being evaluated and also distinguishing between right and left hand images. The evaluation was performed using the testing dataset with the remaining 40% of participants (45 out of 112) and has been undertaken by means of 10-fold cross validation, where 9 folds are used for estimating the classifier model, and the remaining fold for obtaining the performance. This procedure has been carried out 25 times using different random seed numbers (which ensure that fold observations are different on each evaluation) in order to obtain a statistically significant average of the model performance. These results were compared against each other and also with the performance of the linear regression approach.

### 3.5 Machine learning classifiers

The classifiers used in this study were also part of the WEKA environment [32] (version 3.8) successfully used in different fields as a Machine Learning engine [33–35]. Four different machine learning classifiers have been tested as possible candidates to predict the demographic traits. The chosen classifiers cover a range of popular modes of classification: decision trees (J48), probabilistic (Naïve Bayes), support vector machines and logistic regression, and have been selected for complementarity in assessment.

The four classifiers were implemented with their default parameter values (for implementation details please refer to the WEKA documentation [32]).

#### 3.5.1 Decision tree

Decision tree learning is one of the most commonly used algorithms for automatic learning. The decision trees are composed of nodes (which test the value of an attribute), branches (path to follow based on the attribute value) and leaves (which provide the classification of the instance). The decision tree analysed in this work is the C4.5 implementation developed by Quinlan [36], implemented in WEKA as the J48 algorithm.

#### 3.5.2 Support vector machine

Support vector machines were introduced by Cortes & Vapnik [37] in 1995 and have been successfully used in a wide range of different areas. SVM algorithms are based on finding the optimal separating hyperplane that maximizes the margin, in other words, the hyperplane that gives the largest minimum distance to the training examples. The implementation used in this investigation is the LibSVM [38] as an add-on to the WEKA system. The SVM classifier uses a “one-against-one” approach for the multiclass classification, and the kernel type used was the radial basis function.

#### 3.5.3 Multilinear logistic regression

Multilinear logistic regressions [39] is one of the most commonly used tools for discrete data analysis. Multinomial logistic regression is used to predict the probabilities of the different classes analysed given a set of independent variables. It represents a particular solution to the classification problem that assumes that a linear combination of the observed features can be used to determine the probability of each particular outcome of the dependent variable.

#### 3.5.4 Naïve Bayes

The Naïve Bayes classifier [40] is based on the probabilistic Bayes’ rule and is particularly suited when the dimensionality of the inputs is high. In order to reduce the complexity of the high dimensionality, the Naïve Bayes classifier assumes that the effect of the value of a particular feature on a given class is independent of the values of the other predictors. Despite its over-simplified and generally unrealistic assumptions, the Naïve Bayes classifier has been shown to perform remarkably well in a wide range of applications such as text classification [40] and internet traffic identification [41].

### 3.6 Linear regression and categorisation

With the aim of comparing the performance of machine learning classification algorithms with the commonly used linear regression approach, an evaluation of this approach has also been conducted with the SSD data. Linear regression models were created using the three datasets analysed (male, female and whole sample) and both left and right hand data from the training dataset. Their performance was evaluated on the testing dataset using the following methodology:

1. feature selection was performed for linear regression models and the three search directions provided by the “best-first search” algorithm (forward, backward and bi-directional), using all the samples in the dataset,
2. with the selected features, a linear model was created for the prediction of height, weight and foot size,
3. the predictions were categorized into the same 3, 5 and 7 bins detailed in Section 3.3,
4. the accuracy of the binned predictions were calculated.

## 4 Results

In this section, the results of our experimentation are detailed. The large number of evaluations performed in this work (with the combinations of three sample datasets, right and left hand features, four demographic traits, three search directions and five classifiers including linear regression) requires the summarisation of results.

Section 4.1 provides the accuracy rates obtained for sex classification, presenting the results by classifier, search direction and right/left hand. Following the sex prediction results, in Section 4.2 the analysis from the prediction of height, weight and foot size will be discussed. In presenting these results they will be summarised for the different elements of the machine learning classification approach: feature selection direction, classifiers and number of bins. The machine learning classifier results will, furthermore, be compared with the linear regression approach.

### 4.1 Sex classifications results

As sex is a categorical variable with two classes, the binning of variables as applied to other demographical traits is not required. Furthermore, in sex classification only the combined sample dataset is considered. The absence of these two factors (number of bins and sample group) considerably reduced the number of evaluations required. The performance obtained for the sex prediction using the four machine learning classifiers, the three feature selection search directions and both right and left hand is presented in Table 5.

**Table 5:** Summary sex classification accuracy rates.

	Right Hand			Left Hand		
	BA	BI	FO	BA	BI	FO
J48	76.1	85.0	85.0	66.7	71.2	71.2
SVM	76.1	**88.7**	**88.7**	68.8	78.3	78.3
LOG	73.2	81.0	81.0	69.8	84.7	86.0
NB	79.0	79.6	79.6	83.1	**87.8**	**87.8**

Feature Selection search directions:

BA = Backwards, BI = Bidirectional, FO = Forward

Classifiers: J48 = Decision tree, SVM = Support Vector Machine,

LOG = Logistic Regression, NB = Naïve Bayes

The sex classification results show good performance overall with the Support Vector Machine (SVM) classifier producing the best results using forward (or bidirectional) feature selection and right hand images (88.7% success ratio). It can also be seen from the results in Table 5 that right hand features obtained better results than left hand features. The best performance using left hand features was obtained also using Naïve Bayes classifiers, 87.7% success ratio.

The best two classifiers (from right and left hand features) used the following lengths as predictors (Table 6).

**Table 6:** Predictors for sex classifier: right and left hand features.

	Predictors Right Hand		Predictors Left Hand
#	Description	#	Description
1	Wrist Breadth	1	Wrist Breadth
2	Wrist To Little	6	Wrist To Thumb
7	Little Proximal Phalange		

Regarding the feature selection search directions, forward (and bi-directional) direction generally obtained better results. Our analysis showed that a bidirectional search direction generally obtains the same subset of predictors as a forward direction search. The rationale behind this is because both search directions were set to start from an empty feature set.

The results obtained are similar to those identified in other studies, with the exception of [7] where hand shape features and machine learning techniques were used. However, our analysis has shown that the Support Vector Machine (right hand features) and Naïve Bayes (left hand features) classifiers outperforms the commonly used logistic regression approach. A direct comparison of results is not possible due to the use of a different dataset and evaluation techniques across the different articles.

### 4.2 Height, weight and foot size results

The prediction results for the demographic traits of height, weight and foot size have been analysed in terms of the feature selection search direction, the machine learning classifiers, and the number of bins, and performance has been compared with that of the linear regression approach. Considering the possibility of predicting sex from hand features (success rate around 90%), we have also evaluated both machine learning and linear regression approaches for male and female subject subsets.

#### 4.2.1 Feature selection search direction

The “best-first search” feature selection algorithm has three different options for the search direction: backward, forward or bi-directional. These three directions were used in our experiments in combination with: the four different classifiers, the 2 hand feature sets (right and left hand features), the three sample groups (male, female and whole sample), the three binning options (3, 5 and 7) and the three demographic traits. The combinations of all these factors resulted in 648 performances values.

**Fig 3:**
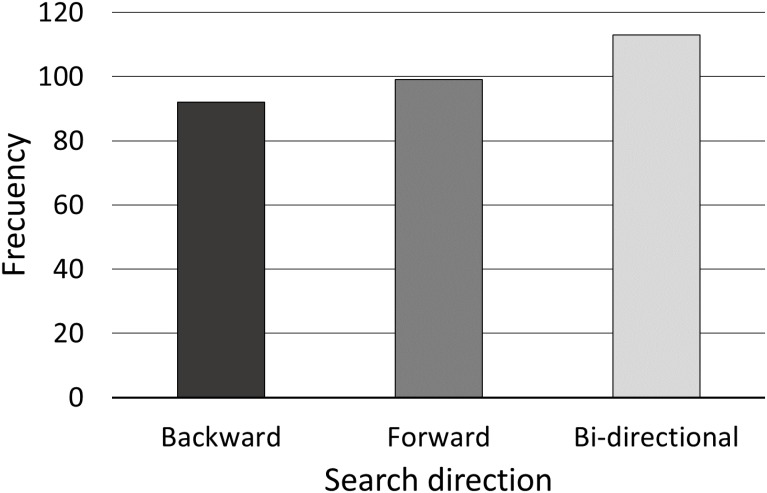
Frequency of best search directions.

For each combination of these five factors (classifiers, hand feature sets, samples, binning and demographics), Fig 3 summarizes how many times each of the three search directions obtained the highest success ratio (when two search directions obtained equal results, both of them were taken into account).

As can be seen, the bi-directional search direction obtained a higher frequency than the forward or backward directions. The bi-directional and forward search directions shared a common starting empty feature subset, however bi-directional has the advantage of allowing both single-attribute additions and deletions, whilst forward direction only allows single additions.

#### 4.2.2 Machine learning classifiers

**Fig 4:**
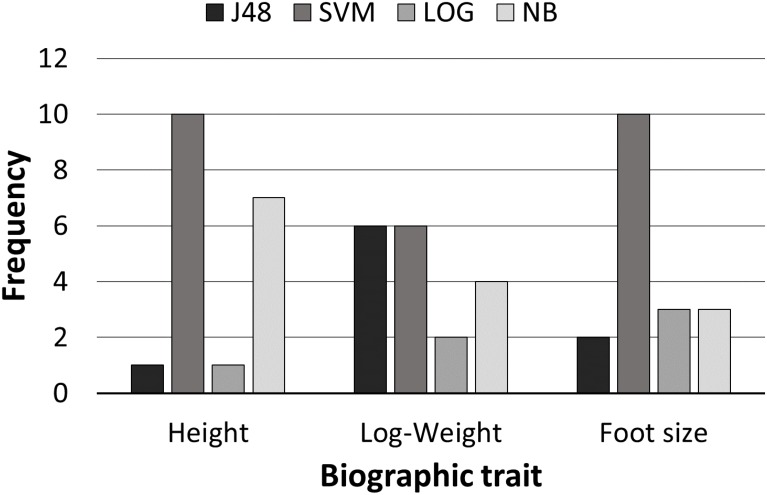
Frequency of best classifiers by demographic trait.

Analysis of the number of times in which a particular classifier obtained the highest performance for each combination of number of bins, group samples and left and right hand feature sets allowed us to assess the relative performance across the techniques. Fig 4 depicts the resulting frequencies for each classifier and each demographic trait. Overall, the Support Vector Machine (SVM) classifier showed the best performance with robustness across all the demographic traits.

#### 4.2.3 Number of bins

**Fig 5:**
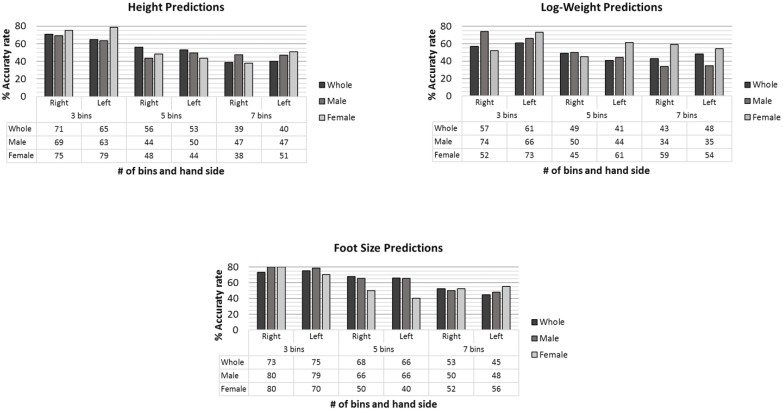
Height, weight and foot size performance comparison for the number of bins, group population and right and left hand.

Assessing the impact of the number of bins for categorisation of demographic traits, the larger the number of bins, the more granularity within the demographic traits categories. Furthermore, a larger number of bins can also imply a better distribution of the sample within those categories. In Fig 5, the success ratios for height, weight and foot size predictions are shown. They are grouped by number of bins and right and left hand, and detailed for each sample group. As expected, the higher the number of bins, the lower the performance. However, the performance drop between 5 and 7 bins may be compensated by the increase in resolution.

In general terms, the demographic trait predictions for 3 bins shows a success rate around 70%, with similar performance for the left and right hand. In some group samples and demographic traits the success ratios are 80%, such as foot size prediction in male and female subjects.

For 5 bins, the accuracy rate reduces to around to 50% for height and weight predictions and around 60% for foot size, showing again similar results between both hands for the three sample groups, although there is a significant decrease on accuracy for the prediction of female foot size. The accuracy rate is around 40% (50% for foot size prediction) when 7 bins are considered, showing equivalent performance within right and left hand assessment and sample groups with the exception of female log-weight prediction which, in this case, shows higher performance than men and whole population.

It is worth noting that the range of these bins is different across the three sample groups. In the case of the whole sample, the range of the values is greater as is the width of each resultant category.

#### 4.2.4 Linear regression

Using the same hand features extracted from the SSD dataset and the associated demographic information, linear regression models have been created for height, weight and foot size. The models used the 21 hand measurements detailed in Table 3 as predictors, and included the four key traits as response variables.

Using the same feature selection algorithm, (BestFirst) and its three search direction options, hand feature subsets have been found for the three demographics, the three sample groups and both right and left hands. The best feature subsets, in terms of adjusted R-squared (Adj R^2^) and root mean squared error (RMSE), was selected from the three search directions, which led to nine linear regression models for each right and left hand features. These linear models are summarized in Table 7.

**Table 7:** Summary of linear regression models.

Demographic	Dataset	Hand	Direction	Predictors ID	Adj. R^2^	RMSE
Height	Whole	Right	backward	5,12,13,14,17,18,19	0.56	6.40
		Left	backward	2,3,5,7,10,12,13,16,18	0.46	7.10
	Male	Right	backward	3,4,11,14,15	0.29	7.05
		Left	backward	1,3,4,5,7,8,9,10,11,12,13,15,16,17,18,21	0.32	6.87
	Female	Right	bi-directional	3,4,14,15	0.40	5.78
		Left	bi-directional	1,2,3,5,6,9,12,16,17,20,21	0.42	5.66
Log-weight	Whole	Right	backward	5,7,9,10,11,12,13,15,21	0.58	0.12
		Left	backward	1,3,4,6,8,9,13,14,16,17,18,20,21	0.66	0.11
	Male	Right	backward	2,4,6,7,8,9,10,11,12,13,19,21	0.34	0.11
		Left	backward	1,3,4,5,6,7,9,10,11,12,13,14,16,17,18,19	0.58	0.09
	Female	Right	backward	1,2,3,4,5,7,8,9,11,12,13,14,16,17,18,19,20	0.52	0.12
		Left	backward	1,4,5,6,7,8,10,12,13,15,16,17,18,20,21	0.60	0.11
Foot Size	Whole	Right	bi-directional	6,15,18,21	0.78	1.12
		Left	backward	1,2,3,4,7,13,15,16,17,18,21	0.73	1.26
	Male	Right	bi-directional	21	0.21	1.40
		Left	bi-directional	1,2,3,4,5,6,7,8,10,11,14,15,17,20,21	0.33	1.29
	Female	Right	backward	2,4,9,12,17,20,21	0.60	0.82
		Left	bi-directional	2,3,5,10,13,16,17,19,21	0.67	0.75

RMSE in cm for height, log-kg for log-weight and UK foot sizes for foot size

From the linear model statistic provided in Table 7, it can be observed that the models obtained from right and left hand features have similar degree of fitness (Adj R^2^). However, the hand features selected from right and left hand models are significantly different. This could be explained by the feature selection algorithm and the high correlation between hand features. In order to compare the accuracy across demographic traits, RMSE values have been normalized by the demographic mean values, obtaining adimensional values suitable for comparison. Height can be predicted within 3.8% of accuracy, the log-weight 2.6% and the foot size 15.5%. The poor prediction of foot size may be explained due to the intrinsic shoe size discretisation.

In S3 and S4 Figs in the Supporting Information Section, the scatter plots of the real data (grey dots) and the fitted data (black lines) are provided for both right and left hands and for the different datasets created from the combination of each demographic trait (height, log-weight and foot size) and each sample group (whole, male and female).

#### 4.2.5 Comparison between linear regression and machine learning classification predictions

**Fig 6:**
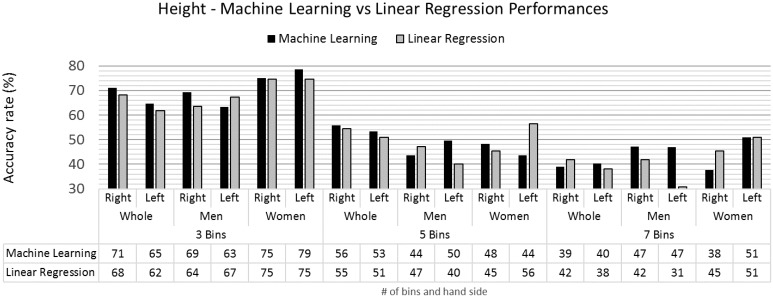
Height accuracy rates for the machine learning and linear regression models.

Using the linear regression models described in Section 4.2.4, the model predictions for height, log-weight and foot size were calculated. These predictions were categorised within the same bins system used for the machine learning approach (Section 3.3). Using these categorised predictions, the binning accuracy rates were calculated for the different combination of demographic traits, number of bins and group samples. Fig 6 compares the success rates obtained from the machine learning classification models with the performance of the linear regression model predictions for height estimation.

The performance of the machine learning classifiers are generally better than the linear regression models, especially when the number of bins is lower.

**Fig 7:**
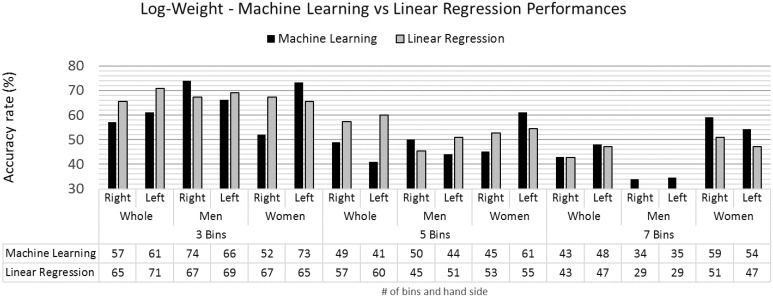
Log-weight accuracy rates for the machine learning and linear regression models.

The performances of log-weight models for machine learning classification and linear regression are presented in Fig 7. For this demographic, machine learning classifiers and linear regression models provide the best performance for the same number of combinations amongst bins and group populations.

**Fig 8:**
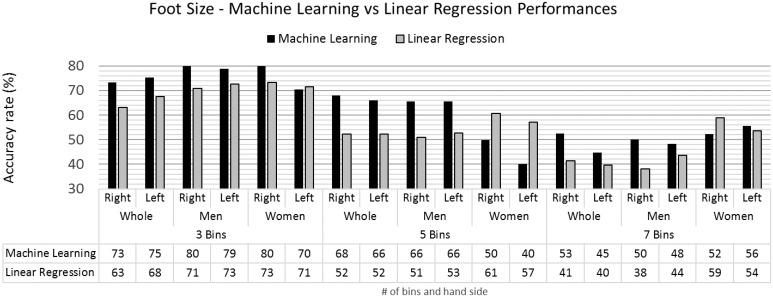
Foot size accuracy rates for the machine learning and linear regression models.

For foot size, Fig 8, the performance of machine learning classification models is generally higher than that obtained from linear regression. These results can be explained by the fact that foot size is itself an ordinal variable, and therefore, more suitable for machine learning approaches.

### 4.3 Comparison with previous work

As previously mentioned, direct comparisons with previous work is difficult due to the use of different population groups, different dataset sizes and different evaluation methodologies. Furthermore, the datasets used in previous studies are either not publicly or easily available, which leads to an inability to reproduce and compare with published results.

However, it is possible to implement previous approaches and obtain their accuracy using our publicly available dataset, and compare the performance of our new machine learning methods. These results will allow us to better understand the challenges of soft-biometric prediction from hand images.

In the following sections, the results of implementing previous approaches for sex and height predictions based on hand features are presented. The implementation of the foot length prediction has not been possible due to the differences in the data collected. In [27] both the hand and foot length have been obtained from the participants, whilst in our analysis shoe sizes were collected instead. For weight prediction, no previous studies were found by the authors of this work.

In order to implement these previous approaches, some approximation had been used. For those studies based on metacarpals and phalanges lengths [18, 25], bones lengths have been approximated with our feature sets. The feature set proposed in this work is based on the skeletal hand structure, and therefore is a reasonable approximation. Some other studies are based on hand length [9, 10, 25]. This length have been approximated using the length from wrist to ring finger plus the sum of the three index phalange lengths. Finally, in the work using hand breadth length [9], the implementation has been based on the “Width Palm Knuckles” hand feature as an alternative.

#### 4.3.1 Sex prediction

Table 8 presents the results of previous published models for sex predictions based on hand features using the SSD dataset. Compared with Table 1, where the published results were summarised, both studies [8, 18] present around 10% drop in terms of accuracy rate. The inferior results show the dependency with regard the dataset used for feature selection and evaluation.

**Table 8:** Sex prediction comparison.

First Author	Year	Ref.	Features	Hand	Accuracy Rate
T. Case	2007	[18]	Index Proximal Phalange	Right	73.7
			Ring Proximal Phalange		
			Little Intermediate Phalange		
			Index Distal Phalange		
			Middle Distal Phalange		
			Ring Proximal Phalange		
			Ring Proximal Phalange		
			Ring Proximal Phalange		
			Index Proximal Phalange	Left	69.4
			Ring Proximal Phalange		
			Little Intermediate Phalange		
			Index Distal Phalange		
			Middle Distal Phalange		
			Ring Proximal Phalange		
			Ring Proximal Phalange		
			Ring Proximal Phalange		
T. Kanchan	2009	[8]	Width Palm Knuckles	Right	78.0
			Width Palm Knuckles	Left	74.3
Our Implementation	2016		Wrist To Little	Right	88.7
			Wrist Breadth		
			Little Proximal Phalange		
			Wrist Breadth	Left	87.8
			Wrist To Thumb		

The results achieved in this paper improve both the published and the implemented accuracy rates from [8, 18]. The work presented by Khanpetch et al. [15] could not be implemented as it was based on metacarpal features such as bone widths and diameters, which could not be approximated from our feature set. The work presented by Amayeh et al. [7] could not be replicated due to the complexity of the extracted features. Finally, the work presented by Jee et al. [20] could not be implemented due to the use of maximum hand thickness feature, which it is not included in our hand feature set.

The significant performance improvement between our results and the implementations of previous works have to be moderated by the fact that our implementation is based on feature selection using this specific dataset. These results show the dependency of these analyses on the dataset used and highlight the need for public datasets in order to be able to fairly compare different approaches. The public availability of the hand subcorpus dataset is one of the aims of this study in order to encourage further research in this field and allow direct comparisons.

#### 4.3.2 Height prediction

Tables 9 to 11 shows the results obtained with previously published models for height prediction using the SSD dataset for the three different population groups: male, female and the whole population.

**Table 9:** Male population height prediction comparison.

First Author	Year	Ref.	Features	Adj. R^2^	RMSE (cm)
S. G. Sanli	2005	[10]	Left Hand Length	0.23	7.32
A.K. Agnihotri	2008	[9]	Left Hand Length	0.16	7.65
			Width Palm Knuckles		
S. R. Habib	2010	[25]	Right Hand Length	0.22	7.32
Our Implementation	2016		Wrist Breadth	0.32	6.87
			Wrist To Ring		
			Wrist To Middle		
			Wrist To Index		
			Little Proximal Phalange		
			Little Intermediate Phalange		
			Little Distal Phalange		
			Ring Intermediate Phalange		
			Ring Distal Phalange		
			Middle Proximal Phalange		
			Middle Distal Phalange		
			Index Proximal Phalange		
			Index Intermediate Phalange		
			Index Distal Phalange		
			Width Palm Knuckles		

**Table 10:** Female population height prediction comparison.

First Author	Year	Ref.	Features	Adj. R^2^	RMSE (cm)
S. G. Sanli	2005	[10]	Left Hand Length	0.44	5.54
A.K. Agnihotri	2008	[9]	Left Hand Length	0.40	5.74
			Width Palm Knuckles		
S. R. Habib	2010	[25]	Left Hand Length	0.45	5.49
			Right Hand Length		
			Right Index Intermediate Phalange		
Our Implementation	2016		Wrist Breadth	0.42	5.66
			Wrist To Little		
			Wrist To Ring		
			Wrist To Index		
			Wrist To Thumb		
			Little Distal Phalange		
			Index Proximal Phalange		
			Index Intermediate Phalange		
			Thumb Distal Phalange		
			Width Palm Knuckles		
			Ring Distal Phalange		

**Table 11:** Whole population height prediction comparison.

First Author	Year	Ref.	Features	Adj. R^2^	RMSE (cm)
S. G. Sanli	2005	[10]	Left Hand Length	0.54	6.53
Our Implementation	2016		Wrist To Index	0.56	6.40
			Ring Distal Phalange		
			Middle Proximal Phalange		
			Middle Intermediate Phalange		
			Index Intermediate Phalange		
			Index Distal Phalange		
			Thumb Proximal Phalange		

As with sex prediction, the multilinear regression models obtained using previously published models are not as accurate a fit as reported in [9, 10, 25]. These results confirm, yet again, how ethnicity can have a significant impact on the hand feature selection and height prediction models.

The proposed height prediction models for the male and whole populations groups present a better adjusted R-squared and lower RMSE than the models implemented from previous work, except for female height prediction. A disadvantage of our implementations is that these better fit values are obtained with more complex models in terms of the features included as predictors.

Similar precautions as in sex results comparison have to be taken when comparing our results for height prediction with previous studies. The feature selection step and the model estimation are highly dependent on the dataset used. Again, the presence of public datasets from different populations will allow an exploration of height (and other soft-biometric traits) prediction models, and will eventually bring more robustness against population-variation.

## 5 Conclusions and future work

This study aimed to explore the relationship between hand measurements and a range of demographics features. In particular we have investigated how height, weight, sex and foot size can be predicted from 21 hand measurements using common implementations of machine learning classifiers and, for comparison, linear/logistic regression techniques. Within our machine learning classification analysis we have examined the division of demographic data using a series of ‘bins’ thereby allowing the prediction of a data range from the hand measurements. This range of predictions has value in biometric deployments by enabling the significant advantages that accompany the use of soft-biometrics.

Our experiments have shown that machine learning classification typically out-performs linear (logistic) regression for the prediction of these four demographic traits based on bin assessment. Although the linear regression approach has the added advantage of being able to predict a continuous range of demographic outcome, the binned soft-biometric prediction of the machine learning classification algorithms may be an interesting option to enhanced biometric identification/verification systems by narrowing search space or improving the certainty (or the time required) to reach a decision.

Our results are in line with previous experimentation (allowing for variances in test datasets). Furthermore, we have contributed a novel relationship between subject weight and hand dimension. We have also shown that, if the sex of a subject is used as internal meta-data, we can use this to enhance the prediction of weight, height and foot-size even further by using a model specific to male or female subjects.

It should be noted that we have employed our machine learning classifiers without optimisation, leading to the real possibility of being able to improve on our results using these methodologies. Future work could involve enhanced feature selection algorithms, fine-tuning of algorithm parameters, analysis of the dependency of performance with respect feature subsets and utilisation of an ensemble of machine learning algorithms in order to further enhance classification success rates. The public availability of the SSD hand dataset will allow further research in these areas. Moreover, the authors consider that making this dataset publicly available will encourage other researchers to do so and enable the creation of multi-ethnicity datasets. This could boost the creation of more accurate and comprehensive soft-biometric prediction models. Another advantage of having larger datasets is that researchers will be able to use larger machine learning training sets that could further improve the accuracy rate of this approach.

Our results are directly applicable to both the biometric and forensic communities. In the former, relationships between these four demographic traits can be used as soft-biometrics to reduce search-space or otherwise provide additional inferred information about the subject undergoing authentication. Results prove that the accuracy of the predictions, specifically in the case of 3 bins (89% for sex prediction, around 70% of success ratio for height, 80% for foot size, and 65% for weight), make them all suitable for use as soft-biometric features. Furthermore, their combined use as soft-biometrics can lead to much greater improvements than their individual use. Within the forensics community, a deeper understanding of the physical characteristics of a subject in relation to their demographics adds evidence when establishing identity. As part of the SuperIdentity project [42], these relationships and predictive abilities will form part of an holistic model of identity linking physical, cyber, demographic and psychological attributes with application to a wide range of end-uses and communities.

## Supporting Information

S1 Fig Demographic trait distributions for the whole, male and female populations. (EPS)

S2 Fig Bin frequencies for the combinations of height, weight and foot size demographics and whole, male and female populations. (EPS)

S3 Fig Right hand linear regression models for height, weight and foot size and whole, men and women groups. (EPS)

S4 Fig Left hand linear regression models for height, weight and foot size and whole, men and women groups. (EPS)
